# Deadly combination of Vaping-lnduced lung injury and Influenza: case report

**DOI:** 10.1186/s13000-020-00998-w

**Published:** 2020-07-09

**Authors:** Bindu H. Akkanti, Rahat Hussain, Manish K. Patel, Jayeshkumar A. Patel, Kha Dinh, Bihong Zhao, Shaimaa Elzamly, Kevin Pelicon, Klemen Petek, Ismael A. Salas de Armas, Mehmet Akay, Biswajit Kar, Igor D. Gregoric, L. Maximilian Buja

**Affiliations:** 1 Divisions of Pulmonary, Critical Care and Sleep Medicine, McGovern Medical School, Houston, TX USA; 2 Advanced Cardio-Pulmonary Therapeutics and Transplantation, McGovern Medical School, Houston, TX USA; 3 Department of Pathology and Laboratory Medicine, McGovern Medical School, The University of Texas Health Science Center at Houston (UTHealth), 6431 Fannin St. MSB 2.276, Houston, TX 77030 USA

**Keywords:** Vaping, Influenza, Acute respiratory distress syndrome (ARDS), Diffuse alveolar damage (DAD), Case report

## Abstract

**Background:**

E-cigarette and vaping use-associated acute lung injury (EVALI) has been recently recognized as a complication in individuals who use vaping devices. Another consideration is that EVALI may have an adverse influence on the outcome of intercurrent respiratory infections. We document this deadly combination in the case of a young man who had EVALI and simultaneous Influenza-A infection leading to severe Acute Respiratory Distress Syndrome (ARDS).

**Case presentation:**

A 27-year-old male with a history of tobacco and vaping use was admitted to hospital after two weeks of flu-like symptoms, diarrhea and vomiting. A chest x-ray was consistent with multifocal pneumonia, and microbiological tests were positive for Influenza-A and methicillin-sensitive Staphalacoccus aureus (MSSA). Bronchoscopy provided evidence of acute inhalational injury. After admission, he acutely decompensated with severe hypoxia and hypotension; he required intubation, sedation and vasopressors. He developed sepsis with acute kidney failure, liver failure, biventricular systolic dysfunction and severe rhabdomyolysis. He was placed on veno-venous (VV) extracorporeal membrane oxygenation (ECMO) initially and later changed to Veno-Arterial (VA) ECMO. Nevertheless, the patient continued to deteriorate, and he expired two weeks after admission.

**Conclusion:**

This case documents that EVALI can act as a major factor leading a respiratory infection to progress into severe ARDS with a fatal outcome.

## Introduction

Electronic cigarettes, or vaping devices, have become increasingly popular in recent years. The short- and long-term health effects of e-cigarette use are still poorly understood but hundreds of cases of e-cigarette or vaping product use-associated lung injury (EVALI) have been reported [1]. In the USA, the number of e-cigarette related emergency department visits has been slowly increasing since 2017 [2]. The use of vaping devices is especially prevalent in the age group of 11 to 34-year-olds [1]. While the mechanism of lung injury in patients who vape is under investigation at the state and federal levels, vitamin E acetate, a chemical used to dilute tetrahydrocannabinol (THC) oil in vape cartridges, has emerged as one of the likely causes of EVALI [3–7]. In January 2020, the FDA finalized a policy that will prioritize enforcement against certain e-cigarette flavors and manufacturers of products that are targeted to minors or have failed to take adequate measures to prevent minors’ access [8]. The aim is to significantly reduce the prevalence of vaping among children while still providing a viable alternative for current combustible tobacco smokers, seeking to cut back [4]. We present a fatal case of EVALI complicated by Influenza-related Acute Respiratory Distress Syndrome (ARDS) (Table 1). This case documents that EVALI can be a major predisposing and contributing factor causing a respiratory infection to progress into severe ARDS with a fatal outcome.

**Table 1:** Timeline

**Fig. 1:**
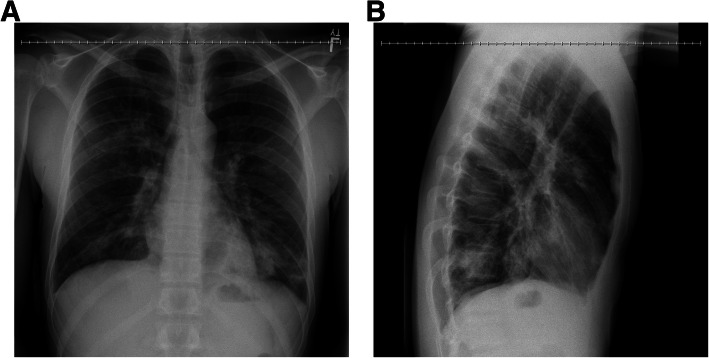
Chest x-ray on admission; PA and lateral view. Patchy infiltrates can be seen in both lower lobes and the right upper lobe

Patient is a 27-year-old African American male. Patient has a 2-year history of cigarette tobacco use and vaping of tetrahydrocannabinol (THC). Patient presents to an emergency room with a 2-week history of nonproductive cough, subjective fever, rhinorrhea, chills, myalgia, diarrhea, and vomiting. He is diagnosed with a viral upper respiratory tract infection and is discharged. Two days later, in mid-December 2019, because of worsening symptoms, patient is admitted to an outside hospital. Infection work up is positive for Influenza-A by nasal swab, and sputum grows methicillin-sensitive *Staphylococcus aureus* (MSSA) a few days later. Initial chest x-ray reveals patchy infiltrates of the right upper and bilateral lower lobes that are consistent with multifocal pneumonia (Fig. 1). Patient’s respiratory status declines rapidly, and he is transferred to the intensive care unit (ICU) and intubated for respiratory failure. Bronchoscopy shows evidence of damage to the trachea and upper bronchi, likely due to vaping. Treatment is started with vitamin C, thiamine, hydrocortisone and multiple antibiotics (vancomycin, cefepime, azithromycin and doxycycline) for concern of sepsis as well as oseltamivir for Influenza A. Patient’s clinical condition continues to deteriorate, and he is transferred for a higher level of care. Patient is treated with V-V ECMO, VA-ECMO and IABP. Patient develops acute renal failure, liver failure, biventricular systolic dysfunction and rhabdomyolysis. Patient expires after a total hospital course of 2 weeks. Autopsy is performed and reveals severe DAD and lipid-laden macrophages consistent with lipoid pneumonia.	

*Abbreviations*: *ECMO* extracorporeal membrane oxygenator, *DAD* diffuse alveolar damage, *IABP* intra-aortic balloon pump, *V-A* veno-arterial, *V-V* veno-venous

## Case presentation

### History

A 27-year-old African American male with a history of cigarette tobacco use (2 years) and vaping of tetrahydrocannabinol (THC) presented with two weeks of nonproductive cough, subjective fever, rhinorrhea, chills, myalgia, diarrhea, and vomiting. He had reported to an outside emergency room 2 days prior and was diagnosed with a viral upper respiratory tract infection and discharged. Despite over-the-counter medication and fluids, he experienced no improvement of his symptoms. However, he continued to have fever, chills, myalgia, rhinorrhea, and dry cough, and so he returned to an outside hospital in mid-December, 2019.

On admission, he was tachypneic and tachycardic with leukocytosis and lymphopenia. An initial chest x-ray revealed patchy infiltrates of the right upper and bilateral lower lobes that were consistent with multifocal pneumonia (Fig. 1). A respiratory pathogen panel was positive for Influenza A (real time polymerase chain reaction test on nasal swab), and, in a few days later, his sputum grew methicillin-sensitive *Staphylococcus aureus* (MSSA). Tests for other pathogens were negative. Upon admission to the intensive care unit (ICU), his initial complete blood count (CBC) was remarkable for white blood cell count (WBC) of 9.8 k/gL with 92.4% neutrophils and 3.8% lymphocytes and a platelet count of 97 k/ gL. His basic metabolic panel (BMP) was remarkable for creatinine of 2.34 mg/dL, AST of 343u/L, ALT of 144u/L, lactic acid of 9.8 mMol/L, and procalcitonin of 26.86 ng/mL. Ferritin was elevated at 5181 ng/mL during the hospitalization.

### Differential diagnosis

The patient’s clinical presentation, imaging and laboratory results were consistent with acute pneumonia. The microbiological workup confirmed Influenza A and MSSA. The patient’s frequent use of e-cigarettes was a possible predisposing factor for EVALI and vaping-related lipoid pneumonia. Additionally, given significant elevation in ferritin with subsequent severe leukopenia, lymphopenia and thrombocytopenia, acquired hemophagocytic lymphohistiocytosis (HLH) was considered in the differential diagnosis.

### Treatment

**Fig. 2:**
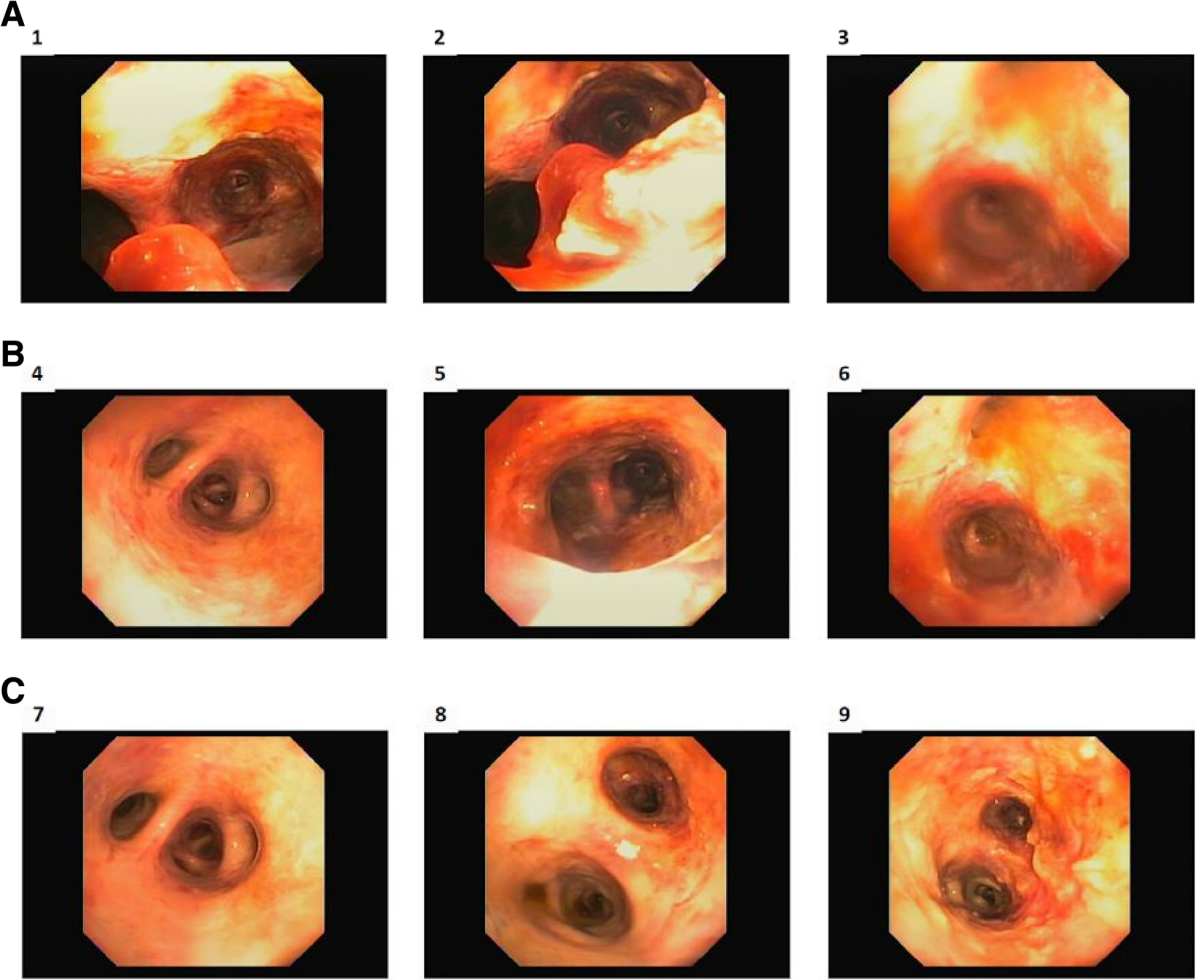
Bronchoscopy on admission provided evaluation of the tracheobronchial tree. Sequential images from proximal to distal show diffusely edematous, denuded main airways indicative of inhalational injury. The very distal subsegments were spared

His respiratory status declined rapidly, and he was transferred to the intensive care unit (ICU) and intubated for respiratory failure. The patient underwent a bronchoscopy which revealed copious airway secretions and a thick, sloughing, erythematous mucosa. Bronchoalveolar lavage (BAL) yielded degenerated material with benign pulmonary elements and acute inflammation, but no signs of malignancy. The bronchoscopy revealed severely denuded airways throughout the lungs consistent with an inhalational injury likely due to vaping (see bronchoscopic images, Fig. 2).

**Fig. 3:**
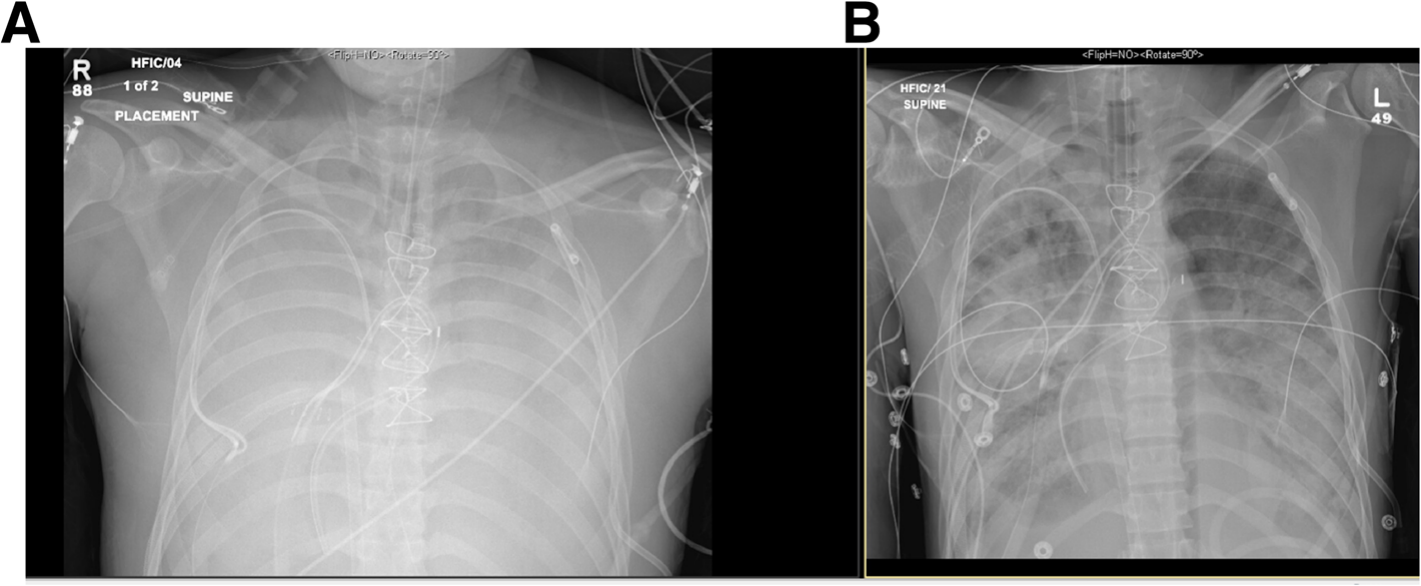
**a** Chest x-ray on admission to ICU one day after initial CXR at admission showing diffuse infiltrates with loss of lung volumes. **b** Improvement in aeration noted in four days after cannulation with ECMO

He had no clinical history of inhalational injury other than the vaping exposure. He had severe progressive refractory respiratory failure despite maximal ventilatory strategies with a progressive increase in airway pressure and dropping Pa02/Fi02 ratio. At this time, he became hypotensive and was vasopressor dependent. Treatment was started with vitamin C, thiamine, hydrocortisone and multiple antibiotics (vancomycin, cefepime, azithromycin and doxycycline) for concern of sepsis as well as oseltamivir for Influenza A. Despite antibiotic treatment, his hypoxic respiratory failure further progressed and he required aggressive ventilatory support with a positive end-expiratory pressure (PEEP) of up to 30. The patient’s Pa02/Fi02 ratio was only 37 when the extracorporeal membrane oxygenation (ECMO) team was contacted and a bilateral femoral veno-venous (V-V) ECMO was placed at the spoke institution as part of our mobile ECMO program and patient was brought to our hub institution. After the procedure he was stable enough to be transferred to our facility. Despite the V-V ECMO, the patient required multiple vasopressors and although his lactic acid decreased initially, there was a progressive need for escalatory support and patient was converted patient from V-V ECMO to veno-arterial (V-A) ECMO. Given severe sepsis with shock, the peripheral V-A ECMO was deemed to be insufficient to provide hemodynamic support, and he was subsequently placed on central V-A ECMO to improve end-organ function. The patient’s clinical course was complicated by acute kidney failure for which he required dialysis, liver failure and encephalopathy which was likely secondary to his metabolic derailment and sepsis. He also experienced multiple episodes of atrial fibrillation with rapid ventricular response for which he was successfully cardioverted three times. A transthoracic echocardiogram showed a severely dilated right ventricle as well as biventricular systolic dysfunction. Due to a worsening pulmonary edema, he was emergently placed on an intra-aortic balloon pump (IABP). Although the CXR showed subsequent improvement in aeration with improvement in minute ventilation on the ventilator (Fig. 3), the patient also developed severe limb ischemia with skin mottling and ecchymoses on his extremities, trunk, nose and ears. Sepsis and cytokine storm with severe vasoplegia requiring vasopressors were determined to the ongoing issue. Severely diminished tibial flow and thrombi in the bilateral posterior tibial arteries was seen on vascular ultrasound and out of concern for compartment syndrome the patient underwent a fasciotomy. During this time, patient was awake, alert and communicative with the family at the bedside. Despite our best efforts, non-viable muscle was seen in all four compartments bilaterally and a bilateral through-knee amputation was performed. Confronted with the patient’s poor prognosis, the family decided to suspend life-supporting measures in favor of transition to comfort care. The patient expired shortly after the IABP and ECMO were turned off. In total, the patient spent two weeks in the hospital. The family consented to an autopsy.

### Autopsy

**Fig. 4:**
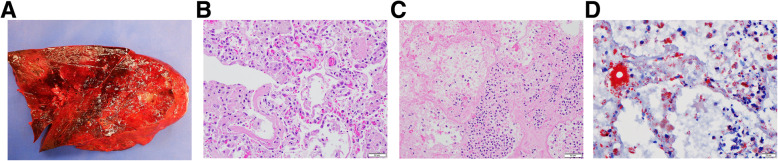
**a** Gross photo of a cut surface of lung, which shows solid, hemorrhagic areas with necrosis. **b** Microscopic photo of diffuse alveolar damage (DAD) in the exudative phase, with hyaline membrane formation, fibrinous and cellular exudate in alveolar spaces. **c** Large area of necrotizing bacterial pneumonia. **d** Special stain Oil Red O, demonstrating lipid containing macrophages in alveolar spaces and alveolar wall

The most important findings in his autopsy are in the lungs, which were grossly solid and extremely heavy (Fig. 4a). No thromboemboli or masses were found in the lungs. The pleural surfaces were dusky and uneven with anthracotic pigment deposition. The pulmonary parenchyma was congested with a mottled appearance due to areas of consolidation, hemorrhage, and necrosis. Histologically, features of diffuse alveolar damage (DAD) were seen (Fig. 4b) with numerous hyaline membranes and alveolar fibrin deposits, together with exuberant necrotizing lobar pneumonia (Fig. 4c). In the non-necrotic lung parenchyma, many foamy histiocytes are present in the alveolar spaces and lipid-containing cells are seen in the alveolar walls, highlighted by special stain Oil-Red O (Fig. 4d). Multifocal, extensive lymphoplasmacytic pericarditis was present, but there was no evidence of myocarditis. Patchy lymphoplasmacytic interstitial infiltrates were found in both kidneys. DAD was considered to be the immediate cause of death of the patient. A lung specimen was sent to the Infectious Disease Laboratory of the Center for Disease Control. The specimen tested positive for influenza virus and negative for SARS-CoV-2 virus.

## Discussion

The critical questions in this case are how and why a young man would die after such a short clinical course which began as an upper respiratory infection. The patient had lymphopenia suggesting he was immunocompromised. Why? By reviewing the patient’s history, we propose that the patient’s vaping with inhalation of a Vitamin E acetate and tetrahydrocannabinol mixture had a major role. In our case, the Oil Red O stain, which stains the lipid droplets in macrophages/ histiocytes, was strongly positive in the sections from the patient’s lungs. This is supporting evidence of vaping, but it is not specific [9–11]. However, acute inhalational injury was documented by video bronchoscopy, and this provided confirmatory evidence of the vaping-induced lung injury [3–7, 12, 13].

Influenza is one of the consistent causes of death attributed to viral pneumonias every year [14]. EVALI due to vaping has been recently recognized as a concern in young patients who use vaping 168 devices [1–8]. Our patient had a complex of influenza-related severe lung injury and rhabdomyolysis further complicated by vaping of a mixture of Vitamin-E and THC. In an experimental murine model, chronic exposure to vapor from electronic nicotine delivery systems (ENDS) or e-cigarettes, independent of nicotine, did not result in pulmonary inflammation or emphysema, but chronic exposure to the vapor did produce aberrant phospholipids in alveolar macrophages and increased surfactant-associated phospholipids in the airways [15]. ENDS vapor exposure downregulated innate immunity against viral pathogens in resident macrophages. Furthermore, ENDS vapor-exposed mice infected with influenza demonstrated enhanced lung inflammation and tissue damage. Altered innate immunity also has been implicated in chronic vaping related tracheobronchial and pulmonary injury in patients [13].

Recent reports have revealed that constituents of E-liquids with Vitamin-E (alpha-tocopherol) acetate (VEA) is the likely culprit in worsening respiratory failure in these patients [16–18]. Vaping is the process of inhaling an aerosol that is created by heating a liquid or wax containing various substances, such as nicotine, cannabinoids causing acute lung injury that can be lethal. Although never officially approved by the FDA, over the past few years, vaping/E-cigarettes has become a popular trend and has been considered “safer” alternative to smoking cigarettes, especially among young adults. The use of E-cigarettes has rapidly increased, and according to the most recent report by CDC released at the end of December 2019, 2506 cases of lung disease and more than 54associated deaths due to EVALI were reported [19]. Initial data shows that most patients use E cigarettes containing tetrahydrocannabinol (THC); some use nicotine while others used a combination of both nicotine and THC. EVALI should be suspected in patients who have a history of vaping or other use of e-cigarette-related products and have a pneumonia-like syndrome, progressive dyspnea, and/or worsening hypoxemia, all of which were found in our patient [20, 21]. The main differential diagnosis for EVALI is community acquired pneumonia (CAP) which can be treatable with antibiotics. The exact mechanism of the lung injury in vaping patients is not fully understood. However, vaping increases the susceptibility of the respiratory tract to both pathogenic and opportunistic infectious agents due to immune dysfunction. In one study, the inflammatory cytokines like Interleukin (IL)-6 and IL-I were highly elevated in e-cigarette users when compared with non-users [21].

Bronchoscopy in our patient showed severely denuded airways as seen in inhalational injury—a finding that has not been previously reported with vaping-related illnesses. Additionally, vaping likely triggered the respiratory failure in this influenza patient—another new finding. Other striking findings under microscopic examination were the relatively diffuse epicardial lymphoplasmacytic infiltrate and patchy interstitial lymphoplasmacytic infiltrate in both kidneys, likely related to viral infection.

Although VV ECMO initially rescued the patient, influenza-related biventricular dysfunction and fatal rhabdomyolysis with CK elevation > 200,000 contributed to the patient’s decompensation.

Influenza associated rhabdomyolysis has been previously reported in case reports in literature [22–31]. Recent review of literature in 2015 revealed twelve case reports with CK levels ranging from 1317 to 1,127,000 with mean value of 206, 908, with survival of 83% [18]. An additional six cases have been reported in the literature since then [25–31]. It is quite plausible that influenza related rhabdomyolysis is much more common and only the severely ill are being reported in the literature. Escalation of his cannulation to central cannulation gave him the best chance at perfusion possible; despite his completely intact neurologic status, efforts were unable to salvage the extremities. We would like to report that based on biomarker profile (lymphopenia, viral pneumonia, progressive ARDS, ferritin elevation), this is a inflammatory profile that is akin to poor survival reported in COVID-19, and this came true in Influenza as expected [32].

COVID-19 disease has many parallels to our patient, but this patient presented in mid-December 2019 well before the first case of COVID-19 in Texas. Analysis of a lung sample by the CDC confirmed the presence of influenza virus and absence of SARS-CoV-2 virus. Nevertheless, with the ongoing epidemic of COVID-19, whose viral infection leads to a similar devastating course as the one described here, physicians should be aware of, and diagnose, vaping-related lung injury when determining treatment and prognosis, and recognize that COVID-19 like illness with cytokine storm, lymphopenia, severe refractory ARDS, and biventricular dysfunction can occur as noted in the past with influenza.

## Conclusion

In conclusion, our case demonstrates that young, previously healthy individuals who indulge in vaping from modified e-cigarettes and develop intercurrent respiratory infection are at risk of developing severe and potentially fatal ARDS with underlying DAD. This case documents that EVALI can act as a major factor leading a respiratory infection to progress into severe ARDS with a fatal outcome.

## Data Availability

All materials described in the manuscript will be freely available to any scientist wishing to use them for non-commercial purposes, without breaching participant confidentiality.

## Abbreviations

ARDS: Acute respiratory distress syndrome
AV: Arterio-venous
COVID-19: Coronavirus infectious disease 2019
DAD: Diffuse alveolar damage
ECMO: Extracorporeal membrane oxygenation
EVALI: E-cigarette and vaping use-associated acute lung injury
IABP: Intra-aortic balloon pump 229
THC: Tetrahydrocannabinol
VV: Veno-venous
