# Correction: Deadly combination of Vaping-lnduced lung injury and influenza: case report

**DOI:** 10.1186/s13000-023-01345-5

**Published:** 2023-05-05

**Authors:** Bindu H. Akkanti, Rahat Hussain, Manish K. Patel, Jayeshkumar A. Patel, Kha Dinh, Bihong Zhao, Shaimaa Elzamly, Kevin Pelicon, Klemen Petek, Ismael A. Salas de Armas, Mehmet Akay, Biswajit Kar, Igor D. Gregoric, L. Maximilian Buja

**Affiliations:** 1 Divisions of Pulmonary, Critical Care and Sleep Medicine, McGovern Medical School, Houston, TX USA; 2 Advanced Cardio-Pulmonary Therapeutics and Transplantation, McGovern Medical School, Houston, TX USA; 3 Department of Pathology and Laboratory Medicine, McGovern Medical School, The University of Texas Health Science Center at Houston (UTHealth), 6431 Fannin St. MSB 2.276, Houston, TX 77030 USA

**Correction: Diagnostic Pathology 15, 83 (2020)**

10.1186/s13000-020-00998-w

Following publication of the original article [1], In this article the title was incorrectly given as “Deadly combination of E-cigarettes has become a popular-lnduced lung injury and Influenza: case report” but should have been “*Deadly combination of Vaping-lnduced lung injury and Influenza: case report*”.

The original article [1] has been updated.

## References

[1] Akkanti BH, Hussain R, Patel MK (2020). Deadly combination of Vaping-lnduced lung injury and influenza: case report. Diagn Pathol.

